# Cell death shapes cancer immunity: spotlighting PANoptosis

**DOI:** 10.1186/s13046-024-03089-6

**Published:** 2024-06-15

**Authors:** Lixia Gao, Chloe Shay, Yong Teng

**Affiliations:** 1 National & Local Joint Engineering Research Center of Targeted and Innovative Therapeutics, College of Pharmacy, Chongqing University of Arts and Sciences, Chongqing, 402160 People’s Republic of China; 2 Wallace H. Coulter Department of Biomedical Engineering, Georgia Institute of Technology and Emory University, Atlanta, GA 30322 USA; 3 Department of Hematology and Medical Oncology, Winship Cancer Institute, Emory University School of Medicine, 201 Dowman Dr, Atlanta, GA 30322 USA

**Keywords:** PANoptosis, PANoptosome, Immunogenic cell death, Cell death forms, Antitumor immunity

## Abstract

PANoptosis represents a novel type of programmed cell death (PCD) with distinctive features that incorporate elements of pyroptosis, apoptosis, and necroptosis. PANoptosis is governed by a newly discovered cytoplasmic multimeric protein complex known as the PANoptosome. Unlike each of these PCD types individually, PANoptosis is still in the early stages of research and warrants further exploration of its specific regulatory mechanisms and primary targets. In this review, we provide a brief overview of the conceptual framework and molecular components of PANoptosis. In addition, we highlight recent advances in the understanding of the molecular mechanisms and therapeutic applications of PANoptosis. By elucidating the complex crosstalk between pyroptosis, apoptosis and necroptosis and summarizing the functional consequences of PANoptosis with a special focus on the tumor immune microenvironment, this review aims to provide a theoretical basis for the potential application of PANoptosis in cancer therapy.

## Background

Cell death is a vital mechanism for preserving the normal functioning of tissues and the overall health of the body. Additionally, its manipulation serves as a fundamental approach in cancer therapy. Cell death can be divided into uncontrollable, accidental cell death (ACD) and regulated cell death (RCD), depending on whether the process is controlled or not [1]. As a type of RCD, programmed cell death (PCD) is intricately linked to various human diseases, particularly cancer [2]. In the past few decades, apoptosis, necroptosis, and pyroptosis were commonly viewed as distinct forms of PCD, and their regulatory mechanisms are the most clearly studied. They not only play an important regulatory role in the initiation, transduction, and execution of cell death, but are also involved in both homeostasis and disease.

**Fig. 1:**
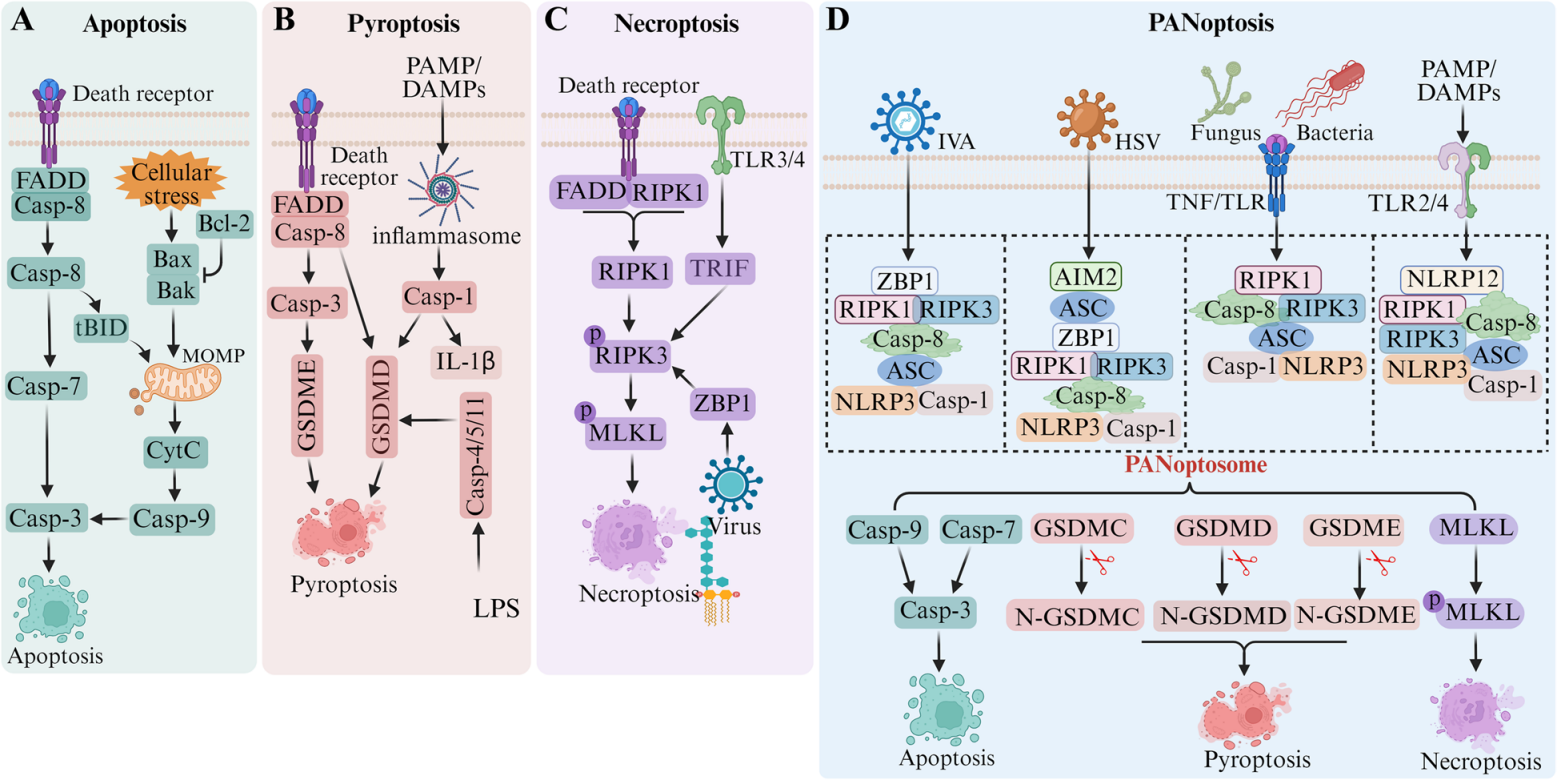
The regulatory mechanisms of apoptosis, pyroptosis, necroptosis and PANoptosis. **A** Apoptosis is divided into intrinsic and extrinsic pathways, which activate the executioner protein caspase-3/7 via the cleavage proteins caspase-9 and caspase-8, respectively. **B** Pyroptosis activates the GSDM family proteins through caspase-1-dependent inflammatosome formation or directly through caspase-4/5/11/8, forming pores in the cell membrane and inducing cell death. **C** Necroptosis phosphorylates MLKL by activating RIPK1 and/or RIPK3 and disrupts the cell membrane to execute death. **D** Under different external stimuli, the composition of the PANoptosome complex is different and thus regulates the downstream PANoptosis pathway by different mechanisms. The PANoptosome is regulated by upstream sensors and molecular signals that assemble into a polymeric complex. These sensors mainly include ZBP1, AIM2, RIPK1 and NLRP12. The signaling molecules mainly include NLRP3, ASC and caspase-1 (pyroptotic proteins), RIPK3 (necroptotic protein), caspase-8 (apoptotic protein)

Although apoptosis, pyroptosis, and necroptosis follow distinct signaling pathways, increasing evidence from numerous studies indicates the existence of mutual crosstalk among them (Fig. 1), ultimately leading to the concept of PANoptosis. PANoptosis as a new type of PCD was proposed in 2019 by Dr. Kanneganti et al. [3]. This mode of death combines the key features of pyroptosis, apoptosis, and necroptosis, which correspond to "P", "A", and "N", respectively, in PANoptosis terminology. PANoptosis is regulated by the PANoptosome complex [4]. This complex is a polymeric complex formed by the cascade regulation of upstream receptors and molecular signals, which acts as an activation platform for downstream molecules and a "master switch" that initiates the three PCD pathways. Upstream molecules of the PANoptosome include Z-DNA-binding protein 1 (ZBP1), absent in melanoma 2 (AIM2), and receptor-interacting serine/threonine protein kinase 1 (RIPK1), which can sense specific stimuli and trigger the assembly of PANoptosis bodies to form the PANoptosome with different sensors and regulatory factors [5].

While the precise molecular mechanisms governing PANoptosis remain unclear, there is evidence suggesting its potential benefits in human diseases, particularly in addressing drug resistance and enhancing tumor immunotherapy [6, 7]. This review discusses the origins of PANoptosis, explores its link to the diverse forms of death pyroptosis, apoptosis and necroptosis, and examines how PANoptosis is regulated in the context of cancer development and treatment. It also highlights the promise of PANoptosis-targeting strategies in cancer therapy.

## The distinct molecular mechanisms of apoptosis, pyroptosis and necroptosis

Apoptosis is a fundamental biological phenomenon of cells that plays a necessary role in multicellular organisms to eliminate unwanted or abnormal cells [8]. It plays an important role in the evolution of organisms, the stability of the internal environment, and the development of multiple systems [9]. In general, apoptosis can be triggered by both internal and external apoptotic signaling pathways (Fig. 1A) [10, 11]. The intrinsic apoptosis pathway, commonly referred to as the mitochondria-dependent pathway, is mainly triggered by mitochondrial outer membrane permeabilization (MOMP) induced by intracellular stressors [12]. MOMP is regulated by Bcl-2 family proteins, which cause the release of cytochrome C (cytc) into mitochondrial intermembrane space. Cytc binds to apoptotic protease activator 1 (APAF-1) and then interacts with pro-caspase-9 to form a complex called the apoptosome. Subsequently mature caspase-9 is able to activate downstream effectors caspase-3 and caspase-7, allowing them to perform their executioner functions. The extrinsic apoptosis pathway is triggered by cell surface death receptors such as FAS and the tumor necrosis factor receptor (TNF-R) family [13]. When the death receptor binds to the corresponding ligand, recruitment of FAS-associated proteins (FADD), RIPK1 and caspase-8 occurs, during which the two death domain effectors, caspase-8 and FADD, interact isotopically. Pro-caspase-8 is automatically processed into mature caspase-8, which cleaves and activates caspase-3 and caspase-7. Thus, caspase-9 and caspase-8 are initiating caspases for endogenous and exogenous apoptotic pathways, respectively, which ultimately complete the apoptotic process via caspase-3 and caspase-7.

Pyroptosis is an inflammatory death pathway mediated by the inflammasome, which can be divided into the canonical (caspase-1-dependent) and non-canonical (caspase-4, -5, and -11-dependent) pathways according to the activation mechanism (Fig. 1B) [14]. There are four main members of the inflammasomes, namely NOD-like receptor family pyrin domain 1 (NLRP1), NOD-like receptor protein 3 (NLRP3), NLR family CARD domain-containing protein 4 (NLRC4) and AIM2 inflammasomes [15, 16]. In the canonical pathway, the inflammasome can sense the external stimulation signal of the cell, recruit and activate caspase-1, which cleaves the peptide segment of the N-terminal active domain of gasdermin D (GSDMD), inducing membrane perforation, cell rupture, and content release, and causing inflammation [17]. In the non-canonical pathway, activated caspase-4, -5 and -11 are stimulated by signals such as bacteria, and then cleave GSDMD to form peptides containing the nitrogen-terminal active domain of GSDMD. In the presence of NLRP3 and ASC, caspase-4 activates caspase-1 to split the preforms of IL-1β and IL-18 to form their mature forms [18]. Membrane perforation and cell rupture are induced, and the cell contents are released, triggering an inflammatory response.

Necroptosis is a programmed lytic cell death pathway, and the key proteins involved in this process include RIPK1, RIPK3, and mixed lineage kinase like (MLKL) (Fig. 1C) [19]. When the activity of caspase 8 is inhibited, RIPK1 and RIPK3 play a key role in TNF-induced necroptosis [20]. RIPK1 and RIPK3 interact to form a necrosome with RIP homotypic interaction motifs (RHIMs), which phosphorylates MLKL protein, and then destroys cell membrane integrity, eventually leading to death. TLR3/4 of the toll-like receptor family can trigger necroptosis if the activity of the protease caspase-8 is compromised [21]. TLR3 and TLR4 can recruit TIR domain containing adapter-inducible interferon B (TRIF) to respond to double-stranded RNA and TLR4 lipopolysaccharide (LPS), respectively. TRIF contains the RHIM domain, which binds RIPK3 and phosphorylates downstream MLKL, leading to necroptosis independent of RIPK1. In addition, ZBP1, a RHIM-containing protein, was also found to induce necroptosis [22].

## Crosstalk between apoptosis, pyroptosis, and necroptosis

**Fig. 2:**
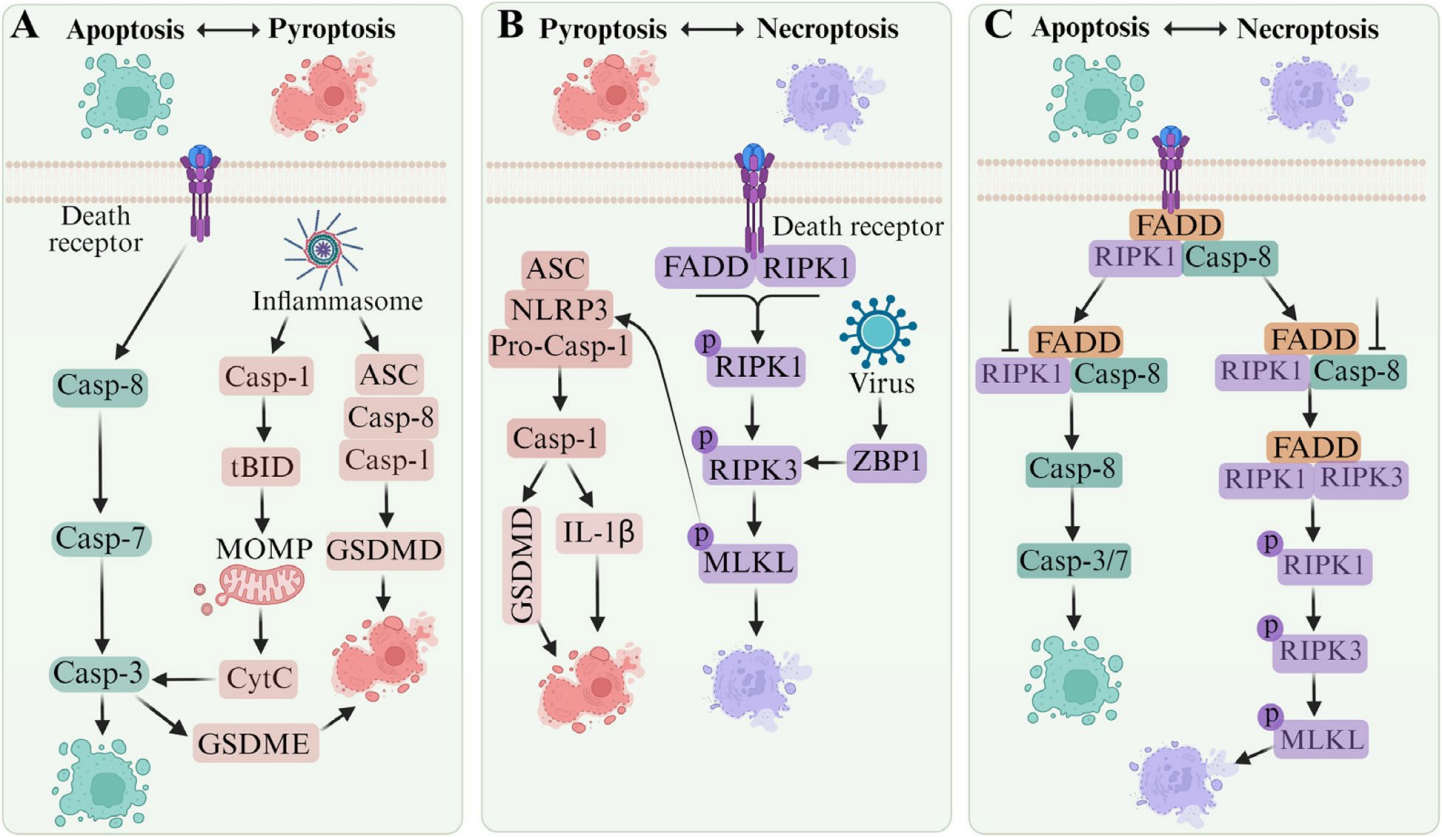
Crosstalk between apoptosis, pyroptosis and necroptosis. **A** The protein caspases link apoptosis and pyroptosis. The inflammasome is involved in the activation of caspase-1, and in addition to cleaving its typical substrate GSDMD, caspase-1 can also mediate the pro-apoptotic protein tBID, which triggers the formation of apoptotic bodies in MOMP. The apoptotic executioner caspase-3 can target the pyrogenic executioner GSDME for proteolysis, resulting in pyrogenic death. **B** The executioner MLKL acts as a bridge between necroptosis and pyroptosis. Regulation of RIPK3 by the exogenous death receptor or ZBP1 leads to phosphorylation of the executioner molecule MLKL, resulting in the formation of pores in cell membranes, including the plasma membrane. In turn, MLKL can activate NLRP3, leading to inflammasome assembly and cleavage of caspase-1 to mediate the formation of GSDMD and IL-1β. **C** Apoptotic and necroptotic are tightly linked, as extrinsic death receptor signaling through the RIPK1/FADD/caspase-8 complex can lead to signal transduction. When RIPKs are inhibited, extrinsic apoptosis occurs via caspase-8 homodimerization. When caspases are inhibited, necroptosis is activated by RIPK1-RIPK3 oligomerization

The complexity of the associations between pyroptosis, apoptosis, and necroptosis suggests that there is a dynamic network of molecular interactions between them, rather than separate, isolated pathways. Not only do they operate with their own distinct regulatory mechanisms, but they also constrain and influence each other to synergistically regulate cellular processes (Fig. 2). Within this framework, there has been a growing body of research exploring the interconnection among apoptosis, pyroptosis, and necroptosis, culminating in the emergence of the concept of PANoptosis.

### Crosstalk between apoptosis and pyroptosis

Pyroptosis and apoptosis are PCD processes that depend on caspases for their execution (Fig. 2A) [23]. Caspase-1 plays a certain regulatory role in both apoptosis and pyroptosis and acts as a bridge between them. When GSDMD is absent, the inflammasome triggers caspase-1 to change mitochondrial MOMP and initiates apoptosis and pyroptosis via caspase-3 and -7, respectively [24]. Researchers have found that caspase-1 and -7 are related, and the regulatory mechanism is that caspase-1 can mediate the activation of endogenous caspase-7 in macrophages. However, the activity of caspase-7 is not completely dependent on caspase-1 [25]. Studies showed that both caspase-3 and -7 were involved in apoptosis, but they have different activation mechanisms in response to microbial stimulation and bacterial infection [26]. Studies also showed that in the absence of downstream GSDMD, caspase-1 cleaves caspase-3 and promotes the occurrence of apoptosis [24]. GSDMD is a protein of great interest between members of the GSDM family as the first identified executor of cell pyroptosis, acting downstream of inflammatory caspase in response to cellular inflammation regulation [27]. By studying the regulatory relationship between GSDMD and caspases, the transition of cells from pyroptosis to apoptosis was uncovered [28, 29].

GSDME/DFNA5 is another member of the GSDMs, and its N-terminal structural domain can form holes in the cell membrane and induce pyroptosis [30]. GSDME can be cleaved by caspase-3, thereby converting noninflammatory apoptosis to pyroptosis in GSDME-expressing cells [30–32]. When GSDME is highly expressed, caspase-3 activity can be activated, leading to cell swelling and rupture, and then pyroptosis. Intriguingly, low expression of GSDME can also induce cell apoptosis [33]. The N-terminus resulting from GSDME cleavage not only triggers pyroptosis but also increases reactive oxygen species (ROS). This increase in ROS levels can reduce mitochondrial membrane potential and release cytochrome C, ultimately culminating in caspase-3-dependent apoptosis [34, 35].

### Crosstalk between pyroptosis and necroptosis

Necroptosis and pyroptosis are often accompanied by the release of potential immunostimulatory molecules (Fig. 2B) [36–38]. Pyroptosis is mainly regulated by the canonical signaling pathway mediated by caspase-1 and the non-canonical signaling pathway mediated by caspase-4, -5 and -11 [39]. Activation of caspase-1 not only disrupts the integrity of cell membranes and causes cell swelling, but also promotes the release of IL-1β and IL-18 [40, 41]. When caspase-4, -5 and -11 are activated, they can cleave GSDMD and lead to pyroptosis. Necroptosis is regulated by RIPK1, RIPK3, and MLKL [42]. The link between necroptosis and pyroptosis comes from a study showing that necrosulfonamide, an inhibitor of MLKL, can suppress GSDMD [43]. In addition, MLKL in the activated state can trigger the NLRP3 inflammasome in a cell-intrinsic manner [44]. Additional studies indicate that necroptosis signaling initiates a RIPK3-MLKL-NLRP3-caspase-1 axis, leading to the maturation of IL-1β [45, 46]. NLRP3 serves as a crucial bridge, linking necroptosis and pyroptosis.

### Crosstalk between apoptosis and necroptosis

It is worth noting that caspase-8 plays a pivotal role in both apoptosis and necroptosis (Fig. 2C) [47]. Caspase-8 not only participates in apoptotic signaling but also regulates necroptotic signaling through the cleavage of RIPK1 and possibly RIPK3 [20, 48]. One study investigated the effects of caspase 8 depletion on apoptosis and necroptosis of TNFα-stimulated ovarian cancer cells [49]. Mechanistically, suppressing NF-κB signaling in ovarian cancer cells changed the effect of TNFα signaling from promoting proliferation to inducing cell death. While cancer cells with high levels of caspase-8 underwent apoptosis, caspase-8 depletion downregulated NF-κB signaling, increased RIPK1 stability, and facilitated necroptotic cell death. These findings have implications for improving anticancer approaches to benefit patients with cancers that express low levels of caspase-8. It is also believed that RIPK1 mutations at Y383F/Y383F promote RIPK1 kinase activation and enhance TNF-induced apoptosis and necroptosis [50]. As a downstream substrate protein of caspase-8, RIPK1 is also involved in apoptosis and necroptosis mediated by caspase-8 [20]. The RIPK1-death domain mediates its dimerization and enzyme activity during necroptosis and RIPK1-dependent apoptosis [51]. In apoptosis-competent cells, complex I (TNFR1 or TNF-RSC) transitions into complex IIa (RIPK1, FADD and caspase-8) to promote apoptosis. When apoptosis is suppressed, necroptosis can be triggered through the assembly of complex IIb, comprising RIPK1, FADD, caspase-8, and RIPK3 (Fig. 2C). This complex facilitates the phosphorylation and oligomerization of MLKL, leading to the initiation of necroptosis. The phosphorylation of RIPK1 plays an important regulatory role in SMYD2 mediating TNF induced apoptosis and necroptosis of colon cancer cells [52]. RIPK3, a member of the receptor interacting protein kinase family, interacts with RIPK1 to form necroptotic complexes, activating MLKL and initiating necroptosis, thereby repressing cancer development [36, 53]. In addition, it has been found that RIPK3 can interact with ZBP1 receptor to mediate necroptosis and PANoptosis [54].

## Historic definitions of PANoptosis

Historically, there has been a consensus that the three types of cell death were distinct processes operating independently within cells (Table 1). Extensive research has elucidated the corresponding regulatory mechanisms and biological functions associated with each (Table 1) [55, 56]. Typically, upon death signal stimulation, caspase-8 can regulate apoptosis mediated by internal and external pathways. Caspase-8 is not only critical in death receptor-mediated apoptosis, but also plays an important role in preventing death receptors, toll-like receptors TLR3 and TLR4, and T-cell receptors from inducing necroptosis [57, 58]. With the deepening of related research, caspase-8 was one of the first discovered bridges between different types of cell death [20, 59]. Several studies revealed that caspase-8 induced cleavage of GSDMs, such as GSDMD and GSDME, the two well-established effectors of pyroptosis [60–62], suggesting the involvement of caspase-8 in the process of apoptosis and pyroptosis. These discoveries provide substantial theoretical underpinnings to shape and articulate the concept of PANoptosis, which was first reported in 2016 and formally introduced in 2019 [3, 63]. PANoptosis emerged from studies exploring the interplay between the inflammasome/pyroptosis and apoptosis and necroptosis. This form of PCD, identified as an inflammatory process, is unique in that it incorporates elements of pyroptosis, apoptosis, and necroptosis, defying a singular explanation by any one of these processes alone [3]. Recently, the study of PANoptosis has been mainly concerned with diseases related to the immune response, cancer, infectious diseases, and others.

**Table 1:**
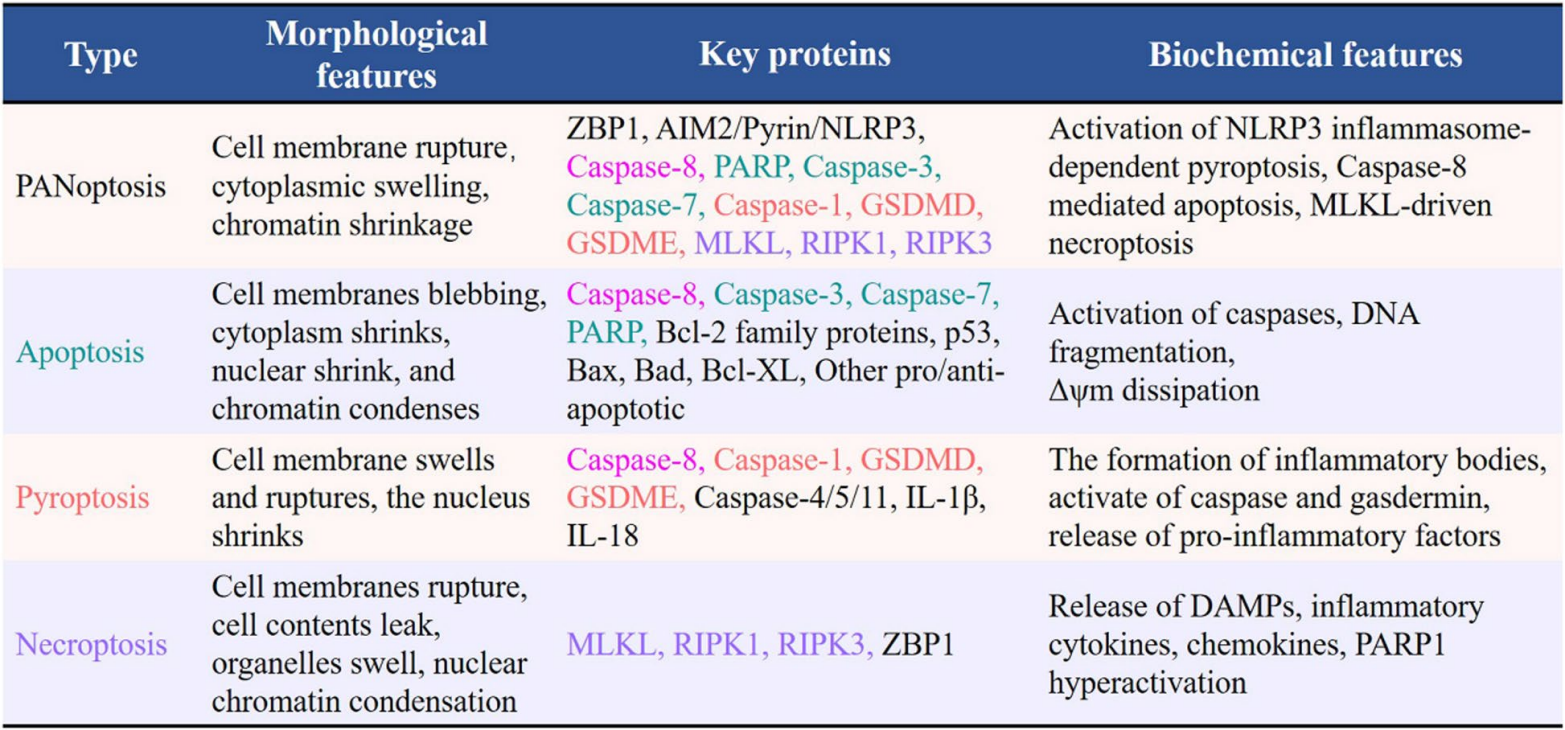
Distinct features and interrelationships between apoptosis, pyroptosis, and necroptosis. Apoptosis, pyroptosis, and necroptosis are the three vertices of the cell death triangle. The reciprocal transformation between them is mainly regulated by intermediary molecules and finally, under certain conditions, PANoptosis occurs

## Evolution and interaction network of the PANoptosome

PANoptosis has been observed in different diseases, such as cytokine storms and cancer [7, 64]. To date, studies have shown that PANoptosis, caused by certain infections and cellular stress, is diverse and the regulatory process is complex [65–67]. This complex regulation is achieved by the PANoptosome. The PANoptosome is a multiprotein complex that provides a molecular scaffold containing key proteins that activate pyroptosis, apoptosis, and necroptosis [55]. The skeleton of the PANoptosome is mainly formed by the interaction of homologous and allotypic domains between proteins. Each PANoptosome comprises three essential components: pathogen-associated molecular patterns (PAMPs), damage-associated molecular patterns (DAMPs), and catalytic effectors or executioners [68]. ZBP1 and RIPK1 have been identified as two upstream molecules of the PANoptosome that can trigger assembly of the PANoptosome and can respond to specific stimuli [69]. Moreover, the proteins involved in regulating the upstream and downstream signaling pathways of the PANoptosome include ASC, NLRP3, caspase-8, RIPK3, caspase-6, transforming growth factor kinase 1 (TAK1) and nucleotide-binding leucine-rich repeat-containing receptor 12 (NLRP12) [70–73]. In other words, the PANoptosome contains key molecules associated with pyroptosis, apoptosis, and necroptosis that enable the activation of these three modes of cell death to carry out a pro-inflammatory cell death process and ultimately PANoptosis.

### The regulatory mechanisms of PANoptosis

PANoptosis is a recently identified mode of cell death typically characterized by preserved cell membrane integrity and cellular swelling, often mediated by inflammasome sensors including members of the NOD-like receptor (NLR) family, the DNA receptor AIM2, and the pyrin receptor (Table 1). External stimuli induce the formation of the PANoptosome, which subsequently initiates the onset of PANoptosis (Fig. 1D). Caspase-8 is a typical cysteine protease encoded by the caspase-8 gene, located on chromosome 2q33-34 [74]. It is a central mediator in the extrinsic apoptotic pathway via FADD or TNFR1 and TRAIL associated via death domain receptors [75–77]. Studies have shown that caspase-8 is involved in the cleavage of GSDM family proteins to induce pyroptosis in endogenous apoptotic pathways. It has been reported that the expression of enzymatically inactive caspase-8 (C362S) can cause embryonic lethality and inflammatory tissue destruction in mice by inducing necroptosis and pyroptosis [78]. As one of the earliest identified bridges between different cell death forms, caspase-8 plays an important regulatory role in the process of PANoptosis under certain conditions [23, 61]. Recently, it was discovered that the metabolite α-ketoglutaric acid (α-KG) triggered pyroptotic cell death by promoting GSDMC cleavage via caspase-8 [79]. This research not only elucidates a pyroptotic pathway associated with metabolites but also unveils a previously unreported central pathway extending from ROS-triggered DR6 endocytosis to caspase-8-mediated cleavage of GSDMC, suggesting potential clinical applications in tumor therapy. Caspase-6, a caspase executor that mediates apoptosis, has been recently identified to play a dual role, not only in the regulation of pyroptosis through GSDMD but also in mediating necroptosis via its influence on MLKL, thereby contributing to its involvement in tumorigenesis and immunotherapy [72].

Increasing evidence suggests that PANoptosis may be related to the tumor immune microenvironment (TIME) and drug resistance in the development of cancer. Here, we mainly introduce the role of PANoptosis in the TIME and review the important role of PANoptosis in tumor immunotherapy and drug resistance.

## PANoptosis and the TIME

The occurrence and development of tumor is the result of interaction and co-evolution between tumor cells and the TIME. The TIME refers to the immune microenvironment around tumor cells, including tumor cells, inflammatory cells, immune cells, various signaling molecules and extracellular matrix [80]. In the realm of immunity, tumor cells have developed diverse anti-immune mechanisms, including downregulation of antigen expression, inhibition of DC antigen presentation capabilities, and suppression of cytotoxic T cell tumor infiltration. Understanding infection and PANoptosis is crucial for developing effective strategies to prevent and control infectious diseases, particularly those caused by highly virulent pathogens. Examples of pathogens that may induce PANoptosis include certain strains of the Ebola virus and some highly virulent strains of influenza [81]. Increasing evidence suggests that PANoptosis may play a role in cancer development and the TIME [82]. Therefore, inducing highly immunogenic PANoptosis within tumors could serve as a promising therapeutic approach. This would activate the cancer innate immune response by DAMPs, ultimately enhancing the antitumor function of antigen-specific T cells.

Cells within the tumor microenvironment (TME) possess the ability to modulate the immunosuppressive milieu, thereby influencing cancer cell proliferation [83]. As one of the most abundant immune cells in tumor invasion, neutrophils can enter tumor tissues to form tumor-associated neutrophils (TANs) [84]. TANs are not only involved in the initiation and development of tumors, but also regulate the TME [85, 86]. Research has identified a tumor-promoting cluster of TANs characterized by increased expression of HMGB1 that may interact with the TME through HMGB1-TIM-3 axis [87]. Moreover, HMGB1-positive TANs were recruited to tumor lesions and closely associated with the pathological grades of primary tumors, facilitating immune evasion via the GATA2/HMGB1/TIM-3 axis. Thus, tumor-promoting TAN clusters with HMGB1 overexpression could serve as a prognostic indicator and gauge immunotherapeutic responses.

The great potential of nanomedicine in the treatment of cancer prompted researchers to study their role in PANoptosis and the TME. It has been reported that an ultrasonic nanomaterial using nano/genetically engineered extracellular vesicles was assembled from ultrasound (US) and PLEP (PFH@Lipo-PpIX@EVs) [88]. The combination of US and PLEP had a remarkable impact on the expression of pyroptotic protein GSDMD, apoptotic protein caspase-3, and necroptotic protein MLKL, compared to the control group. Additionally, the proportion of immunosuppressive regulatory T cells (Tregs) was significantly reduced in the US + PLEP group compared to other groups. This reduction may be attributed to the critical role of PANoptosis in reshaping the immunosuppressive TME. Consequently, US + PLEP nanomedical drugs modulated PANoptosis and bolstered CD8^+^ T cells-mediated antitumor immunity [89]. By analyzing the correlation between PANoptosis-related molecular subtypes and prognostic models and the TIME, researchers aimed to explore the mechanism of PANoptosis in pancreatic cancer [90]. This study found that patients with increased CD8^+^ T cells, monocytes, and naïve B cells were more sensitive to irinotecan, oxaliplatin, and sorafenib, while patients with increased macrophages, activated mast cells, and DCs were more sensitive to erlotinib, selumetinib, and trametinib [90]. Xi et al. analyzed the correlation between GSDMD and CD8^+^ T cell markers through the Cancer Genome Atlas (TCGA) database and found that the expression of GSDMD is positively correlated with CD8^+^ T cell marker levels in tumor samples [91]. GSDMD is required for CD8^+^ T cell cytotoxicity and its deficiency reduced the cytolytic capacity of CD8^+^ T cells. When CD8^+^ T cells were activated, GSDMD was cleaved by caspase-4 and -11. To further clarify the effects of different PCD on the ability of DCs to cross-present and initiate CD8^+^ T cell responses, we constructed apoptosis and necrosis models. Although DAMPs release can trigger an inflammatory response, they do not promote strong cross-priming. Further studies showed that RIPK1-mediated induction of NF-κB and its downstream target genes played a key regulatory role in initiating CD8^+^ T cell adaptive immunity [92]. RIPK3, a critical protein in the necroptosis pathway, plays a significant role in cell death regulation. In cancer cells, its activation relies on the downregulation of TRIM28. This activation of RIPK3 contributes to antitumor immunity by promoting the production of immune-stimulating cytokines within the TME [93]. Furthermore, GSDME has been implicated in the pathogenesis of tumors and other diseases. Studies have shown that GSDME expression promotes the phagocytosis of tumor cells by tumor-associated macrophages and enhances both the quantity and functionality of tumor-infiltrating natural killer cells and CD8^+^ T lymphocytes [94].

By integrating the data from TCGA database and three Gene Expression Omnibus (GEO) databases of clear cell renal cell carcinoma (ccRCC), Wang and colleagues constructed a PANoptosis-related microRNA signature using bioinformatics approaches, revealing the potential significance of a PANoptosis-related microRNA signature in clinicopathological features and tumor immunity [95]. Researchers also constructed a PANscore scoring system to quantitatively assess the PANoptosis pattern in individual gastric cancer (GC) patients. Pan-cancer analysis revealed that high PANscore was associated with low expression of immune checkpoints, high expression of TGF-β, and dense infiltration of CAFs and M2-type macrophages [96]. Hepatocellular carcinoma (HCC) is one of the most common malignant cancer, and it was reported that PANoptosis-related genes and relevant clusters were associated with the survival and immunity of patients. The abundance of immune cells, expression of immune checkpoints, and immunotherapy and chemotherapy were also considered to be associated with the risk score, and the prognosis of a high-risk score was poor [97]. Another study, using bioinformatics analysis of expression data for 19 genes identified in previous studies and clinical data for colon cancer patients in TCGA and GEO databases, revealed that a patient’s survival prediction risk score was associated with immune cell abundance, cancer stem cell (CSC) index, checkpoint expression, and response to immunotherapy and chemotherapy drugs [5]. To clarify the role of PANoptosis in GC, researchers analyzed GC specimens and established molecules through expression patterns of PANoptosis regulators and the immune landscape clusters associated with PANoptosis-related genes (PRGs) and corresponding immune characteristics. In this study, five selected variables demonstrated significant associations with infiltrating immune cells and immune-related pathways [98]. This analytical model may be applicable for assessing the risk and immune response of GC patients. The above studies highlight that the majority of current research involves bioinformatic analysis to identify associations between PANoptosis-related genes and the TIME. Subsequent experimental verification tends to be preliminary and straightforward. However, there is a paucity of studies that delve into detailed molecular mechanism exploration, indicating a potential avenue for future research efforts.

## PANoptosis and antitumor immunity

**Fig. 3:**
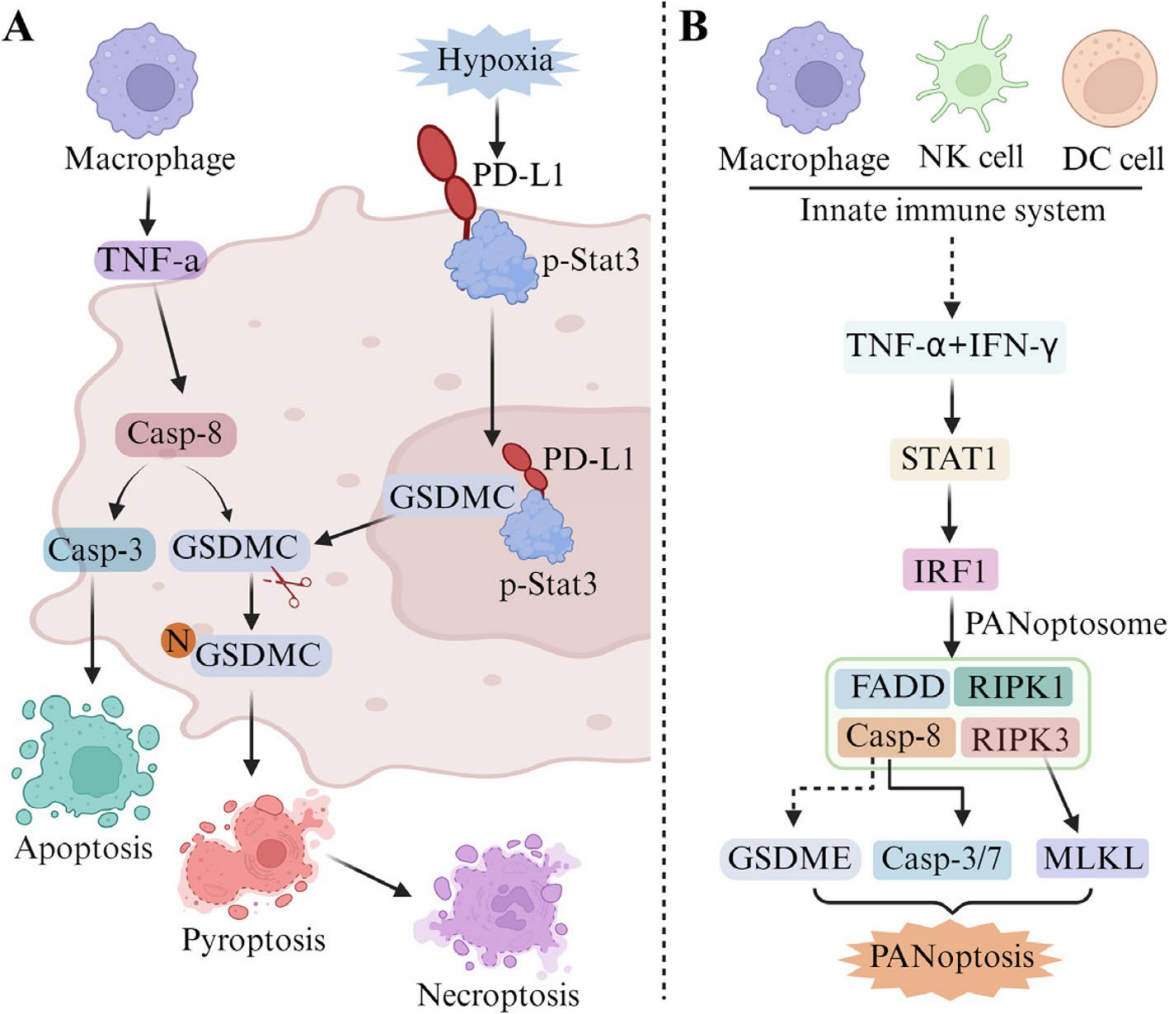
The role of PANoptosis in antitumor immunity. **A** Under hypoxia, p-Stat3 binds to PD-L1 and promotes its nuclear translocation to bind to GSDMC. GSDMC is specifically cleaved by caspase-8 and TNFα stimulation to generate the N-terminal domain of GSDMC, which forms pores in the cell membrane and induces pyroptosis. **B** TNF-α and IFN-γ produced by the intrinsic immune system can induce PANoptosis by activating proteins such as GSDME, c aspase-8, caspase-3, caspase-7, and MLKL

In recent years, the emergence of immunotherapy has prolonged the median survival of certain patients with advanced tumors. Complementing traditional approaches such as surgery, radiotherapy, chemotherapy, and targeted therapy, immunotherapy presents a promising avenue for treating advanced tumors [38, 99]. PANoptosis, with its ability to dynamically regulate PCD through the PANoptosome, stands as a component of the host’s innate immune defense [3, 82]. The PANoptosome acts as a molecular scaffold to regulate the transmission and interaction of its key protein signals in an orderly manner, providing the host with the ability to resist viruses or bacteria [81]. Programmed death molecule ligand-1 (PD-L1) is an important immunosuppressive factor, which can convert TNF-α induced apoptosis of cancer cells into pyroptosis, leading to tumor necrosis [100]. The specific mechanism is that p-Stat3 physically interacts with PD-L1 to promote its nuclear translocation and enhances transcription of the GSDMC gene under low oxygen conditions. GSDMC is specifically cleaved by caspase-8 and TNF-α stimulation to produce the N-terminal domain of GSDMC, which forms pores in the cell membrane and induces pyroptosis (Fig. 3A) [100]. This study demonstrated for the first time that nuclear PD-L1, caspase-8, and GSDMC are essential for macrophage-derived TNFα-induced tumor necrosis. Another study found that TNF-α and IFN-γ were able to induce PANoptosis in 13 different human cancer cell lines from colon, lung, melanoma, and leukemia, through the activation of GSDMD, GSDME, caspase-8, caspase-3, caspase-7 and MLKL (Fig. 3B) [101]. In addition, ultrasound nanomaterials utilizing nano/genetically engineered extracellular vesicles can prompt tumor immunoediting therapy, a strategy that induces tumor highly immunogenic PANoptosis and activates the cancer innate immune system to produce enough antigen-specific T cells to form a protective immune response by repeatedly releasing damage-related molecules [95]. This study suggests that targeting PANoptotic cell death patterns provides a new strategy for overcoming immune escape. In addition, administration of CBL0137, a potent anticancer drug that kills cancer cells by changing their DNA structure, either alone or in combination with LPS induced ZBP1-mediated PANoptosis in macrophages. This process was linked to the generation of Z-DNA in the nucleus independent of NLRP3, likely resulting in assembly of the PANoptosome [102]. Another study analyzed the MSigDB database related to prostate adenocarcinoma PRGs and found that the PANoptosis signature could improve antitumor immunity and promote the infiltration of immune cells [6]. It was also suggested that molecular clustering and prognostic features based on PANoptosis have potential application value in predicting survival and TIME of colon cancer patients [5]. Immunotherapy, exemplified by immune checkpoint inhibitors (ICIs), has achieved remarkable breakthroughs in cancer treatment. However, a significant number of tumors show poor or no response to ICIs, which is often attributed to the insufficient presence of tumor-infiltrating lymphocytes (TILs), thereby limiting the widespread efficacy of ICIs. As the interplay between PANoptosis and tumor immunity, understanding this interaction may reveal the potential utility of targeting PANoptosis to enhance the efficacy of immunotherapy in the treatment of malignant tumors [6, 96].

## PANoptosis and drug resistance

Drug resistance refers to the resistance of tumor cells to antitumor drugs, which, once developed, significantly reduces the therapeutic effect of drugs. At present, the problem of drug resistance has seriously hindered the wide application of antitumor drugs in clinical practice and has brought great challenges to therapeutic impact for patients. Factors contributing to drug resistance in tumor cells typically encompass tumor heterogeneity, the blood–brain barrier, tumor size, the immune system, the TME, and selective treatment pressure, among others [103–105]. As a newly identified PCD mode, the application of PANoptosis in solving drug resistance problems is increasing [7]. Bioinformatics has been employed to scrutinize the potential regulatory roles of long non-coding RNAs (lncRNAs) associated with metastasis and PANoptosis in colorectal adenocarcinoma (COAD). This conclusive analysis revealed a significant correlation of SNHG7, an lncRNA, with COAD prognosis, tumor stage, lymph node metastasis, and drug resistance [106]. Reportedly through the construction of a prognostic signature to predict prognosis and recognize ideal patients for corresponding chemotherapy and immunotherapy, it was suggested that risk score could be a biomarker to predict the response to ICIs, TACE, and sorafenib therapy [107]. There are only few reports on experimental studies that link PANoptosis with drug resistance for antitumor purposes. A study based on the biological function of NFS1 found that its deletion significantly enhanced the sensitivity of colorectal cancer cells to oxaliplatin. In vitro and in vivo results show that NFS1 deficiency synergistically induces PANoptosis with oxaliplatin by increasing the level of intracellular ROS [108].

## Therapeutic applications of PANoptosis in *cancer*

**Fig. 4:**
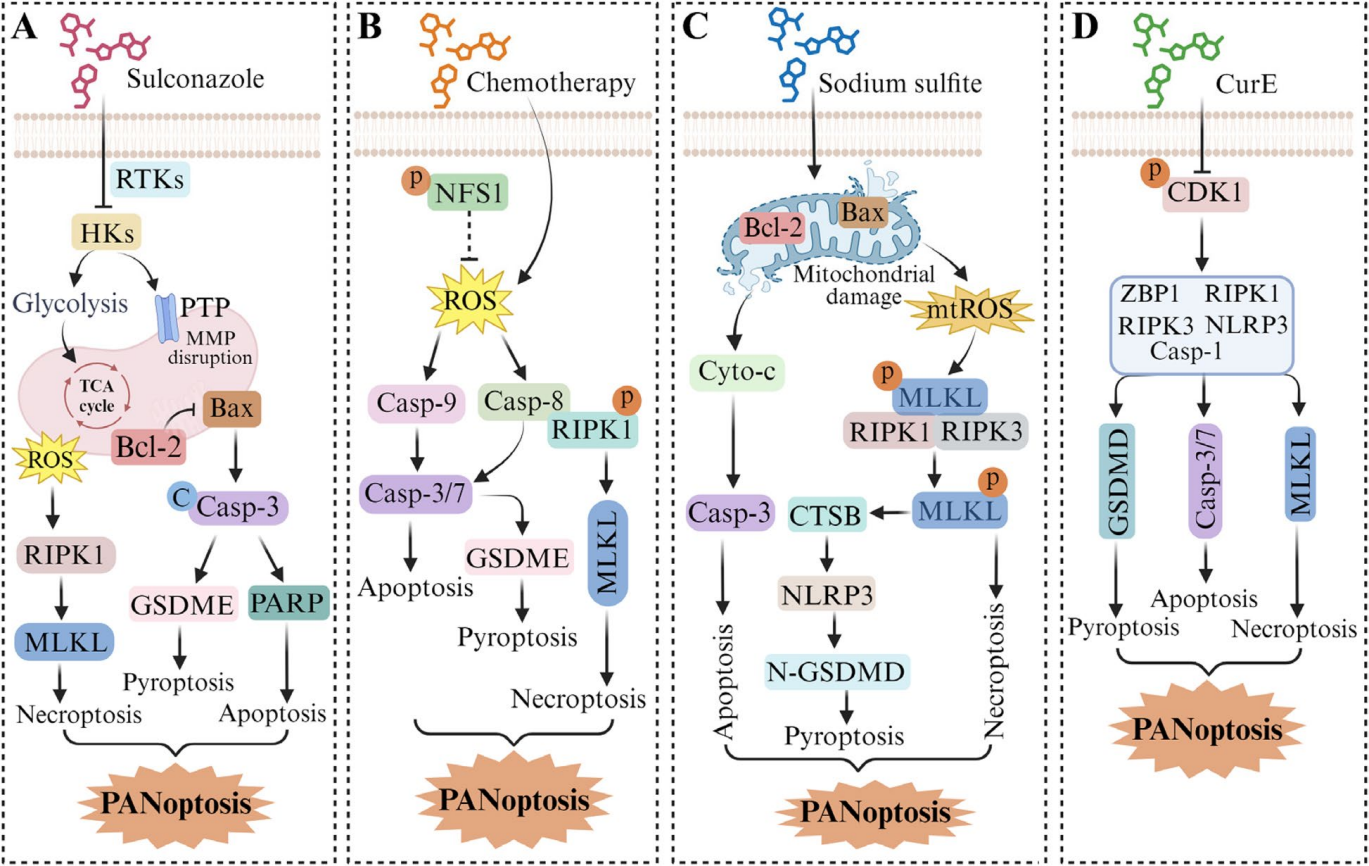
Application of PANoptosis in cancer therapy. **A** Sulconazole shows induction of PANoptosis in esophageal cancer cells. **B** NFS1 deficiency, which acts synergistically with oxaliplatin, induces PANoptosis by increasing intracellular reactive oxygen species (ROS) levels. **C** Sodium sulfite (SS), a common food and drug additive, induces hepatic PANoptosis. **D** CurE induces PANoptosis in ACC cells by binding to the ZBP1 PANoptosome

PANoptosis serves as a critical protective mechanism for cells exposed to various stresses and injuries; thus, activation of PANoptosis may render cancer cells more susceptible to treatments and thereby improve therapeutic outcomes [109]. Currently, PANoptosis is widely acknowledged as an inflammatory form of PCD governed by the PANoptosome complex [15]. Nevertheless, certain chemotherapeutic agents have the ability to trigger inflammasome-independent PANoptosis, thereby increasing cancer cell susceptibility to chemotherapy. Sulconazole, an FDA-approved drug, which has exhibited encouraging anti-tumor properties by impeding proliferation and migration while also inducing PANoptosis in esophageal cancer cells (Fig. 4A) [67]. The mechanism by which sulconazole induces PANoptosis involves mitochondrial oxidative damage, triggering the release of ROS and cytc, as well as prompting abnormal expression of Bcl-2 family proteins, leading to the cleavage of caspase-3 and PARP protein and subsequent apoptosis. Furthermore, activated caspase-3 can cleave GSDME into its N-terminal fragment (N-GSDME), ultimately resulting in pyroptosis. Simultaneously, ROS released by mitochondria can activate RIPK1 and RIPK3 via caspase proteins, leading to the phosphorylation of MLKL and initiation of necroptosis. In addition, findings from both in vitro and in vivo models indicate that deficiency in cysteine sulfase (NFS1) synergistically induces PANoptosis by elevating intracellular ROS levels and activating caspase-8 and caspase-9, respectively, suggesting that inhibition of NFS1 could be used as a promising strategy to improve prognosis of platinum-based treatment in colorectal cancer (Fig. 4B) [108]. Interestingly, the combination of TNF-α and IFN-γ, two pro-inflammatory cell molecules, can also activate the JAK/STAT1/IRF1 signaling axis, inducing nitric oxide production and driving caspase-8/FADD-mediated PANoptosis [101]. IRF1 is closely related to the occurrence of colorectal tumors and is an upstream regulator of PANoptosis, which can induce cell death during tumorigenesis associated with colitis [110]. Kanneganti et al. found that IRF1 can activate the PANoptosome via ZBP1, AIM2, RIPK1, NLRP12 and pyrin. In addition, IRF1 is involved in regulating cell death when inflammasomes together with caspase-8 and RIPK3 form the PANoptosome [111]. Targeting IRF1 represents a therapeutic strategy for inflammatory and infectious diseases and cancer. Sodium sulfite (SS), a common food and drug additive, has been reported to induce hepatic PANoptosis by promoting mitochondrial ROS accumulation and activating the BAX/Bcl-2/caspase-3 pathway to induce apoptosis and the RIPK1/RIPK3/p-MLKL pathway to induce necroptosis (Fig. 4C) [93]. In this study, p-MLKL can also induce the release of cathepsin B (CTSB) through lysosomal membrane perforation, which binds to NLRP3 to induce pyroptosis [112]. Moreover, a significant correlation between high expression of apurinic/apyrimidinic endonuclease 1 (APE1) protein and poor prognosis was observed in NSCLC. Building upon this, researchers conducted a screening of APE1 protein inhibitors from a compound library (NO.0449–0145). As expected, the newly identified APE1 inhibitors exhibited the ability to induce PANoptosis and effectively overcome resistance to cisplatin and erlotinib in NSCLC cells [113]. One research group developed a computational framework to explore the pan-cancer clinical significance of PANoptosis and explore potential targetable biomarkers [114]. This study revealed that elevated expression of PANoptosis-related genes was detrimental in low-grade glioma and kidney renal cell carcinoma but beneficial in melanoma. This study also identified and validated key innate immune biomarkers derived from PANoptosis [114]. The onset and progression of adrenal cortical carcinoma (ACC) has been linked to CDK1 dysfunction. Cucurbitacin E (CurE), a CDK1 inhibitor, was found to regulate ACC cell PANoptosis by binding to the PANoptosome in a ZBP1-dependent manner (Fig. 4D) [115].

## Challenges and prospects

PANoptosis is recognized as an inflammatory cell death mode characterized by a sophisticated regulatory process involving multiple signaling molecules that may contribute to infection, inflammation, and cancer. While the literature on PANoptosis is limited, its initial implication in cancer development and drug resistance suggests its potential as a therapeutic target. At present, most studies have focused on correlating PANoptosis-related genes with cancer development through bioinformatic analysis. Only a small fraction of studies has undergone in vivo and in vitro validation, indicating a potential avenue for future research efforts. There is an urgent need to identify new upstream targets for PANoptosome regulation and to discover chemical agents capable of inducing the PANoptosome. It is also imperative to delve deeper into the intricate molecular mechanisms of PANoptosis in various cancers and identify key regulatory molecules.

In recent years, immunotherapy has shown significant antitumor efficacy, and future research could focus on the development of novel drugs or combination strategies that can induce PANoptosis in cancer cells by mediating RIPK1, caspase-8, caspase-3, MLKL and other pathways to kill tumor cells and reverse the unfavorable TME. While PANoptosis plays a critical role in tumor suppression by stimulating antitumor immunity, some studies have reported that elevated caspase-8 can promote tumor growth and progression [78], suggesting that PANoptosis may also facilitate tumor development under certain conditions. Research in melanoma and prostate cancer has shown that tumor cells can use DNA damage-induced nuclear caspase-8 to override the p53-dependent G2/M cell cycle checkpoint [116]. In addition, high expression of caspase-8 can prevent typical endogenous apoptosis and induce mitosis, thereby promoting tumor progression [116]. This finding is consistent with the observation in HCC, where high nuclear caspase-8 expression was associated with poor survival after partial hepatectomy [117]. Increased caspase-8 activity in various tumor cell lines has also been associated with increased caspase activity, motility and aggressiveness [118, 119]. Therefore, it is of great importance to selectively target caspase-8, to further regulate its role in promoting and inhibiting cancer, and to study its involvement in PANoptosis. It is noteworthy that when targeting PANoptosis as a strategy to enhance antitumor immunity, it is crucial to monitor the activation levels of immune effector cells to prevent the occurrence of cytokine storms.

While there is optimism surrounding the potential of new drugs capable of inducing PANoptosis for cancer treatment, it is important to recognize that despite their theoretical safety and efficacy, there is currently a dearth of patient treatment studies targeting PANoptosis. Research in this area is still in its infancy and no clinical trials have been conducted to date, underscoring the need for further investigation and validation before therapies targeting PANoptosis can be considered for clinical use.

## Conclusion

As PANoptosis has been implicated in various tumors, research into the molecular mechanisms and key regulators of PANoptosis in cancer will fill existing gaps in the field and provide innovative and practical therapeutic approaches for cancer treatment. Although the concept of PANoptosis in cancer therapy is promising, further research is essential to fully elucidate its mechanisms, refine therapeutic strategies, and evaluate efficacy and safety of PANoptosis-related drugs in clinical settings.

## Data Availability

Not applicable.

## Abbreviations

ACC: Adrenal cortical carcinoma
ACD: Accidental cell death
APE1: Apurinic/apyrimidinic endonuclease 1
AIM2: Absent in melanoma 2
APAF-1: Apoptotic protease activator 1
ccRCC: Clear cell renal cell carcinoma
COAD: Colorectal adenocarcinoma
CSC: Cancer stem cell
CTSB: Cathepsin B
CurE: Cucurbitacin E
Cytc: Cytochrome c
DAMPs: Damage-associated molecular patterns
FADD: FAS-associated proteins
GC: Gastric cancer
GEO: Gene expression omnibus
GSDMD: Gasdermin D
HCC: Hepatocellular carcinoma
IAV: Influenza A virus
ICIs: Immune checkpoint inhibitors
lncRNAs: Long non-coding RNAs
LPS: Lipopolysaccharide
MLKL: Mixed lineage kinase domain-like
MOMP: Mitochondrial outer membrane permeabilization
N-GSDME: N-terminal fragment of GSDME
NFS1: Cysteine desulfurase
NLR: NOD-like receptor
NLRC4: NLR family CARD domain-containing protein 4
NLRP1: NOD-like receptor family pyrin domain 1
NLRP3: NOD-like receptor family pyrin domain 3
NLRP12: Nucleotide-binding leucine-rich repeat-containing receptor 12
NSCLC: Non-small cell lung cancer
PAMPs: Pathogen-associated molecular patterns
PCD: Programmed cell death
PD-L1: Programmed death molecule ligand-1
PRGs: PANoptosis-related genes
RCD: Regulated cell death
RHIMs: RIP homotypic interaction motifs
RIPK1: Receptor-interacting serine/threonine protein kinase 1
ROS: Reactive oxygen species
SS: Sodium sulfite
TAK1: Transforming growth factor kinase 1
TANs: Tumor-associated neutrophils
TCGA: The Cancer Genome Atlas
TILs: Tumor-infiltrating lymphocytes
TME: Tumor microenvironment
TIME: Tumor immune microenvironment
TNF: Tumor necrosis factor
TNF-R: Tumor necrosis factor receptor
Tregs: Regulatory T cells
TRIF: TIR domain containing adapter-inducible interferon B
US: Ultrasound
ZBP1: Z-DNA-binding protein 1
